# Mixed Grazing Systems Benefit both Upland Biodiversity and Livestock Production

**DOI:** 10.1371/journal.pone.0089054

**Published:** 2014-02-13

**Authors:** Mariecia D. Fraser, Jon M. Moorby, James E. Vale, Darren M. Evans

**Affiliations:** 1 Institute of Biological, Environmental and Rural Sciences, Aberystwyth University, Aberystwyth, United Kingdom; 2 Department of Biological Sciences, University of Hull, Hull, United Kingdom; Auburn University, United States of America

## Abstract

**Background:**

With world food demand expected to double by 2050, identifying farming systems that benefit both agricultural production and biodiversity is a fundamentally important challenge for the 21^st^ century, but this has to be achieved in a sustainable way. Livestock grazing management directly influences both economic outputs and biodiversity on upland farms while contributing to potentially damaging greenhouse gas emissions, yet no study has attempted to address these impacts simultaneously.

**Methods:**

Using a replicated, landscape-scale field experiment consisting of five management ‘systems’ we tested the effects of progressively altering elements within an upland farming system, viz i) incorporating cattle grazing into an upland sheep system, ii) integrating grazing of semi-natural rough grazing into a mixed grazing system based on improved pasture, iii) altering the stocking ratio within a mixed grazing system, and iv) replacing modern crossbred cattle with a traditional breed. We quantified the impacts on livestock productivity and numbers of birds and butterflies over four years.

**Results, Conclusion and Significance:**

We found that management systems incorporating mixed grazing with cattle improve livestock productivity and reduce methane emissions relative to sheep only systems. Systems that also included semi-natural rough grazing consistently supported more species of birds and butterflies, and it was possible to incorporate bouts of summer grazing of these pastures by cattle to meet habitat management prescriptions without compromising cattle performance overall. We found no evidence that the system incorporating a cattle breed popular as a conservation grazer was any better for bird and butterfly species richness than those based on a mainstream breed, yet methane emissions from such a system were predicted to be higher. We have demonstrated that mixed upland grazing systems not only improve livestock production, but also benefit biodiversity, suggesting a ‘win-win’ solution for farmers and conservationists.

## Introduction

With world food demand expected to more than double by 2050, decisions about how to meet this challenge will have profound effects on wild species and habitats [1], [2]. Livestock grazing is a major driver of land use change worldwide, often leading to the loss of wildlife habitat [3]. In the European Union (EU) increased livestock production has historically been blamed for dramatic changes in biodiversity as a result of overgrazing and habitat modification [4], [5]. Identifying optimal livestock grazing systems that benefit both biodiversity and production is therefore a priority for sustainable agriculture.

The British uplands are internationally important for their unique plant and bird communities, many of which are maintained by agriculture. The high nature conservation value of upland areas in the UK is recognised through the identification of many of the habitats as Priority Habitats in the UK Biodiversity Action Plan (BAP) and their inclusion on the Annex 1 list of habitats under the EU Habitats Directive [6]. Large parts of the uplands are designated as Special Areas of Conservation (SAC) under the Habitats Directive in recognition of these habitats, and are Sites of Special Scientific Interest (SSSIs) under domestic legislation. Restrictions on agricultural activities in upland areas imposed by climate and topography mean systems of ruminant livestock production dominate. High sheep numbers, together with a shift from traditional farming systems of mixed herbivores towards ones dominated by sheep [7], [8], have been implicated in dramatic changes in upland vegetation and bird abundance [5], [9]. Experimental studies support this, showing that intensive sheep grazing pressure adversely affects arthropods [10] and breeding upland birds [11], [12], whereas low intensity, mixed grazing is beneficial.

While decoupling of EU subsidies from agricultural production in 2003 together with other long-term social trends in hill-farming communities led to declines in livestock numbers in the uplands, particularly within Severely Disadvantaged Areas [13], [14], further reforms to the Common Agricultural Policy are imminent. The concept of sustainable agricultural intensification, defined as “producing more output from the same area of land while reducing the negative environmental impacts and at the same time increasing contributions to natural capital and the flow of environmental services” [1], has particular resonance for upland areas. Concomitant information on livestock productivity and biodiversity gains or losses under different grazing scenarios is, however, lacking. There is a similar dearth of information regarding the extent to which management decisions influence the environmental impact of upland grazing systems via livestock greenhouse gas (GHG) emissions. Structural carbohydrates such as cellulose and hemicellulose ferment at a slower rate than non-structural carbohydrates such as sugars, fructans and starch, and yield more methane per unit of substrate digested [15], and so ruminants grazing poorer quality grassland would be expected to yield a greater volume of methane. In 2010, agriculture was the source of around 44% of total UK emissions of methane, and the majority of this came from livestock enteric sources [16]. As part of efforts to meet global commitments relating to climate change, including the Kyoto Protocol, the UK Committee on Climate Change has set an intended GHG emission reduction budget of 42% in 2020 relative to 1990 figures.

In this study we use a replicated, landscape-scale field experiment consisting of five ‘systems’ to assess the impact of management options on 1) livestock performance; 2) two common indicators of environmental change, birds and butterflies [17], [18]; and 3) enteric methane emissions. This is the first time these measures have been carried out simultaneously on a grazed ecosystem. The experiment was designed to test the effects of progressively altering elements within an upland farming system, *viz*: i) incorporating cattle grazing into a sheep-only system, ii) integrating use of semi-natural rough grazings (SNRG) into a mixed grazing system based on improved pasture, iii) altering the stocking ratio within a mixed grazing system, and iv) replacing modern crossbred cattle with a traditional breed. Our prediction was that mixed grazing scenarios present opportunities for achieving both improved production efficiency and greater habitat diversity through the exploitation of between-species differences in foraging behaviour. The extent of the benefits realised were expected to be dependent on whether the grazing strategies of the cattle and sheep were complementary or competitive under different management options.

## Methods

### Ethics Statement

All animal work was approved by the IBERS Local Ethical Review Group. All stock were managed by experienced stockmen in accordance with the Welfare of Farmed Animals (Wales) Regulations 2007, and the farm as a whole had Farm Assured Welsh Livestock accreditation. The conditions under which the animals were studied were designed to be as similar as possible to those used in commercial livestock production systems, and all stock were assessed daily for health and well-being. The research was conducted on one of IBERS own research farms. All pastures were managed in accordance with EU standards of good agricultural and environmental condition (GAECs). Two of the paddocks used were designated as Sites of Special Scientific Interest, and these were managed in accordance with existing grazing prescriptions imposed by the associated regulatory authority for Wales. The field studies did not involve endangered or protected species.

### Experimental Design

An experiment consisting of five grazing treatments (systems) replicated twice was set up at the Bronydd Mawr Research Centre, Powys, Wales (51°37′N 03°38′W). The five systems were: 1) sheep-only, grazing improved permanent pasture (PP) (S-PP), 2) sheep plus Limousin cross cattle stocked at a ratio of 6∶1 grazing PP (S/C6L-PP), 3) sheep plus Limousin cross cattle stocked at a ratio of 6∶1 on PP, with cattle removed to SNRG for approximately 10 weeks from late June (S/C6L-SN), 4) sheep plus Limousin cross cattle stocked at a ratio of 12∶1 on PP, with cattle removed to SNRG for 10 weeks (S/C12L-SN), and 5) sheep plus Belted Galloway cattle stocked at a ratio of 6∶1 on PP, with cattle removed to SNRG for 10 weeks (S/C6BG-SN). Data collection ran for four years from 2005.

The total experimental area covered over 43 ha of improved pasture and 24 ha of *Molinia caerulea*-dominated SNRG. Individual plot sizes on the PP were 2.25 ha for system 1; 4.75 ha for system 2; 4.125 ha for systems 3 and 5; and 6.375 ha for system 4. Plots included land allocation sufficient to harvest enough silage to feed the stock grazing them through the winter and were designed to give an overall annual stocking rate of 1.6 livestock units ha^−1^ yr^−1^ on PP within each system [19]. The SNRG plots were 4 ha in size.

### Sward Management and Sampling

The PP swards were dominated by perennial ryegrass (*Lolium perenne*) and had a white clover (*Trifolium repens*) content of less than 5%. Unsown grasses were mainly meadow grasses (*Poa* spp.) (31%) and bents (*Agrostis* spp.) (8%), with smaller amounts of Yorkshire fog (*Holcus lanatus*) (3%) and fescue (*Festuca* spp.) (1%). Regular sward measurements taken throughout each growing season found no evidence of between-system differences in sward height or sward biomass on these plots [19]. Plot areas allocated to silage production were closed up at the beginning of May, with the crop harvested around mid June (weather permitting). All PP plots received fertiliser at a rate of 50 kg N ha^−1^ in early spring. A second dressing of 80 kg N ha^−1^, 32 kg P_2_O_5_ ha^−1^ and 45 kg K_2_O ha^−1^ was applied to the silage area at the time of close-up.

At least two-thirds of each of the six areas of *Molinia*-dominated SNRG grazed on systems 3–5 was classified as Purple Moorgrass and Rush Pastures Priority Habitat. Additional grassland habitat types recorded as part of the overall mosaic within the plots were Fen Priority Habitat and Lowland Hay Meadow Priority, plus the Broad Habitats of Neutral Grassland, Acid Grassland, Dwarf Shrub Heath and Dense Bracken [20]. Two of the paddocks had been designated as Sites of Special Scientific Interest, and the SNRG areas were grazed by cattle in accordance with previous management prescriptions put in place by the relevant statutory body (the Countryside Council for Wales). Grazing on these began when there was sufficient biomass to sustain the cattle and ceased when utilisation of the current season’s growth of *M. caerulea* reached 50% [21]. Both SSSIs were classified by CCW staff as being in ‘favourable condition’ during the experiment and following its completion.

Herbage samples were collected from the PP plots by cutting the material within a 14 cm × 144 cm quadrat to ground level using electric shears every 4 weeks. Separate cuts were taken from the areas of PP within each replicate system that were grazed/ensiled or grazed only. The number taken was determined using the formula max(6,ceiling(5*area)), and ranged from 6 on the grazed/silage areas for system 1, to 17 on the grazed only areas of system 4. Representative sub-samples of the material cut from each plot were bulked on a plot basis. Samples for analysis to determine sward chemical composition on the SNRG areas were collected every four weeks, to coincide with quadrat sampling on the improved pasture. One bulked sample per plot was collected using mechanical shears. Digestibility of organic matter in the DM (DOMD) was determined on all samples according to the two-stage method of Tilley and Terry [22], adapted for the ANKOM DAISY^II^ 220 incubator system (ANKOM Technology Corporation, Fairport, NY, USA).

### Livestock and Management

Full details of the stock management protocols can be found in [19]. Briefly, the sheep used on all systems were Beulah Speckled Face ewes that were bred to Suffolk rams. They were selected from the main Bronydd Mawr flock based on uniformity of live weight and body condition score (BCS), and stock allocation to plots was balanced for ewe live weight, litter size, lamb live weight and lamb sex. Turnout of sheep began annually in April, and the plots were stocked to give a lamb to ewe ratio of 1.5∶1. Following weaning of the lambs they grazed the silage aftermaths as a priority. Lambs were removed from experiment for slaughter when they weighed over 36 kg and had reached a body condition score equivalent to fat class score 3L [23]. Any lambs remaining on the experimental plots at the end of September were removed from the experiment, and considered sold on as store lambs. Once any remaining lambs had been removed at the end of September the silage area was opened up, giving the ewes access to the entire plot. Cattle were allocated to treatments at the start of grazing according to the age, live weight and BCS of the dam, and the age, live weight and sex of the offspring. Cattle grazing ran from turnout in May. During the post-weaning period the cows and calves grazed the aftermath pastures along with the lambs, with this regime commencing once the cows had returned from the SNRG areas on systems 3–5. The cattle were removed from the plots in early October and moved to winter accommodation, at which time the calves were weaned, removed from the experiment, and considered sold on. The live weights of all stock were recorded regularly throughout the grazing season [19]. Animals were re-allocated to treatments at the beginning of each growing season. Data were collected annually from 288 ewes, 432 lambs, 24 suckler cows and 24 calves.

### Bird and Butterfly Surveys

Bird surveys were carried out weekly by the same observer (JV) throughout the year using a whole-area search method based on the Common Bird Census [24]. On each occasion the species and number of birds interacting with each experimental plot was recorded by the observer walking pre-defined transects. Data collected between April (spring) and March (winter) were pooled to obtain species richness values for each year, e.g. April 2005 to March 2006 = 2005 etc. Butterfly surveys were also carried out weekly using a modified version of the United Kingdom Butterfly Monitoring Scheme (UKBMS). Butterflies observed within 5 m of defined transect lines were identified and recorded from May to September, but only on days which were calm, sunny and with a temperature >12°C.

### Estimating Enteric Methane Emissions

Methane emissions were estimated on a group basis assuming 6.5% of gross energy (GE) intake was lost as methane [25] and gross energy intake was estimated from energy requirements of the cattle and sheep according to [26]. The GE density of methane used was 55.65 MJ kg^−1^, and feed GE density was assumed to be 18.8 MJ kg^−1^ DM [26]. Pre-weaning, energy intake from milk received by suckling calves and lambs was assumed to equal the milk energy produced by lactating cows or ewes, and this was assumed not to contribute to methane emissions. The metabolisability of feed GE at maintenance (q_m_) was calculated from sward sample metabolisable energy (ME) values, with ME densities of forages being calculated as 0.157 × DOMD [26]. Energy requirements for maintenance and growth were estimated from mean live weight and live weight change respectively, with mean scaling factors (C2) of 1.225 and 0.925 for LimX and BG calves respectively, to account for mixed sex groups and differences in the maturing age of the breeds. Milk energy yields of ewes were estimated assuming a milk fat concentration of 70 g/kg and production for an average of 1.5 lambs, and assuming a stage of lactation based on mean lambing dates. Similarly, milk energy yields of the cows were estimated from mean calving dates, and assumed a milk fat concentration of 36 g kg^−1^.

### Data Analyses

The effect of management system on animal performance was investigated with plot as the experimental unit. Livestock data for each of the four years were initially analysed separately using ANOVA with pre-defined contrasts (Genstat 12; VSN International Ltd, Hemel Hempstead, UK), before being combined using meta-analysis methods as described by Whitehead [27]. Generalized Linear Models with Poisson error structures and log link functions were used to investigate the effects of management system, replicate and year (including all possible interactions) on bird and butterfly species richness. The best models for birds and butterflies respectively were selected using Akaike’s information criterion (AIC). Bird and butterfly species densities were also used as response variables in modified models to account for differences in system area (i.e. log_10_ (number of species)/log_10_ (area in ha)). Furthermore we contrasted model outputs with and without species detected in SNRG. Bird and butterfly analysis was carried out in R version 2.13.0 [28].

## Results

### Animal Performance

Output in terms of total lamb and calf liveweight gain per system were calculated (Table 1). Given that all lambs and calves were removed from the systems by the beginning of October, this equates to the annual production figures. The BG calves were smaller than their LimX equivalents, in keeping with what would be expected of a native breed type, and this was reflected in the figures for total calf gain (system *F*_4,35_ = 490.86, *P*<0.001). There was also a significant effect of year on the results obtained (year *F*_3,32_ = 48.25, *P*<0.001), which declined in the second year of the experiment, and again in the third. Pasture type also influenced total calf gain, which was higher for the system based on improved pasture only than those where summer grazing of SNRG was incorporated. However, when the figures were adjusted to take into account system differences in PP land area requirements the pattern changed, and total calf output for the LimX cattle was similar for PP only and the combined PP/SNRG system. The greater number of sheep on the S/C12L SN plots affected the total weight gain results, and the pattern of system-related differences was different when output was adjusted to take into account differences in the area of PP utilised (Table 1). Collective lamb and calf live weight gain was strongly influenced by management system (system *F*_4,35_ = 78.18, *P*<0.001) (Table 1). The highest overall gain was recorded on the S/C6L SN system, with the S/C12L SN similar to that of the S/C6L PP. A year effect on total output was also identified which reflected the year-to-year variation in calf output.

**Table 1:** Effects of upland farming system on livestock output.

	System						F prob.		
	S PP	S/C6L PP	S/C6L SN	S/C12L SN	S/C6BG SN	s.e.d.	S	Y	S × Y
*Per plot (kg)*									
Total calf gain	0	592	549	549	399	36.0	<0.001	<0.001	<0.01
Total lamb gain	320	338	343	677	346	18.3	<0.001	<0.001	ns
*Per hectare (kg ha* ^−*1*^ * PP)*									
Total calf output	0	125	133	86	97	7.7	<0.001	<0.001	<0.05
Total lamb output	142	71	83	106	84	4.4	<0.001	<0.01	ns
Total system output	142	196	216	192	181	9.7	<0.001	<0.001	ns

Where S = management system, Y = year.

S PP = sheep only grazing permanent pasture; S/C6L PP = sheep and Limousin cross cattle grazing permanent pasture at a ratio of 6∶1; S/C6L SN = sheep and Limousin cross cattle grazing permanent pasture at a ratio of 6∶1 with cattle removed to semi-natural vegetation for 10 weeks; S/C12L SN = sheep and Limousin cross cattle grazing permanent pasture at a ratio of 12∶1 with cattle removed to semi-natural vegetation for 10 weeks; and S/C6BG SN = sheep and Belted Galloway cattle grazing permanent pasture at a ratio of 6∶1 with cattle removed to semi-natural vegetation for 10 weeks.

### Estimated Enteric Methane Emissions

Estimated enteric methane emissions per ewe and lamb unit were similar on all systems during the pre-weaning period (Table 2), but treatment differences were detected during the post-weaning phase (system *F*_4,35_ = 3.66, *P*<0.05). When estimated total emissions across the summer grazing period were expressed relative to the growth rates achieved (i.e. as emissions intensities) there was also a significant treatment difference (system *F*_4,35_ = 21.95, *P*<0.001), with the animals on the sheep only system producing the greatest amount of methane per unit liveweight gain.

**Table 2:** Effects of upland farming system on estimated enteric methane emissions of cattle and sheep (where pre-weaning = from turnout in April to weaning in July; post weaning = from weaning to the end of September; early summer = from turnout onto semi-natural rough grazing (SNRG) to return to permanent pasture (PP); late summer = from return to PP until removal for weaning and housing at the beginning of October).

	System						F prob.		
	S PP	S/C6L PP	S/C6L SN	S/C12L SN	S/C6BG SN	s.e.d.	S	Y	S × Y
*Sheep*									
Sheep pre-weaning (g (ewe+lambs)^−1^ d^−1^)	101	94	92	93	90	7.4	ns	<0.001	ns
Sheep post weaning (g (ewe+lambs)^−1^ d^−1^)	83	82	92	90	87	7.8	<0.05	<0.001	ns
Sheep (g kg^−1^ lamb lwt gain)	318	278	300	282	286	18.8	<0.001	<0.01	ns
Cattle									
Early summer grazing (g (cow+calf)^−1^ d^−1^)	–	519	506	520	443	27.2	<0.001	<0.001	<0.01
Late summer grazing (g (cow+calf)^−1^ d^−1^)	–	611	589	551	435	36.9	<0.001	ns	ns
Cattle (g kg^−1^ calf lwt gain)	–	402	438	432	497	35.5	<0.05	<0.001	<0.05
*Combined output*									
Sheep (kg ha^−1^ PP)	62.15	27.96	34.04	21.01	32.59	5.293	<0.001	<0.001	<0.001
Cattle (kg ha^−1^ PP)	–	50.12	57.13	36.77	47.07	5.245	<0.001	<0.001	<0.001
Total per system (kg ha^−1^ PP)	62.15	78.08	91.18	78.78	79.67	5.366	<0.001	<0.001	<0.001
Total per kg output (g kg^−1^ lwt gain ha^−1^ PP)	438	398	425	410	443	30.0	<0.05	ns	ns

Values are for the entire summer grazing period unless otherwise stated. For treatment details see Table 1.

Where S = management system, Y = year.

The early summer grazing period for the cattle ran from the time of turnout onto the SNRG to their return to the PP in August/September. There was some influence of system on estimated enteric methane emissions from the suckler cows at this time (system *F*_4,35_ = 20.72, *P*<0.001), with the output calculated as being lower from BG cows and their calves than from the LimX cattle. The BG cattle also had the lowest emission rate during the late summer period when the cattle were all grazing PP. However, the LimX cattle were estimated to produce less methane per unit of calf growth (system *F*_4,35_ = 4.60, *P*<0.05), and were therefore more efficient converters of pasture to product.

The total amount of methane predicted to be produced by the sheep and the cattle within each system reflected the number of each species present (Table 2). When these figures were combined, system was found to have a significant impact on the total amount of methane estimated to have been emitted, with the sheep-only system producing less methane per unit area than the mixed systems. However, once productivity was also taken into account a different pattern of differences emerged (system *F*_4,35_ = 3.29, *P*<0.05), with lower emission intensities (g methane per kg live weight gain per ha) being associated with the mixed grazing systems incorporating the LimX cattle.

### Bird and Butterfly Species Richness and Abundance

**Figure 1:**
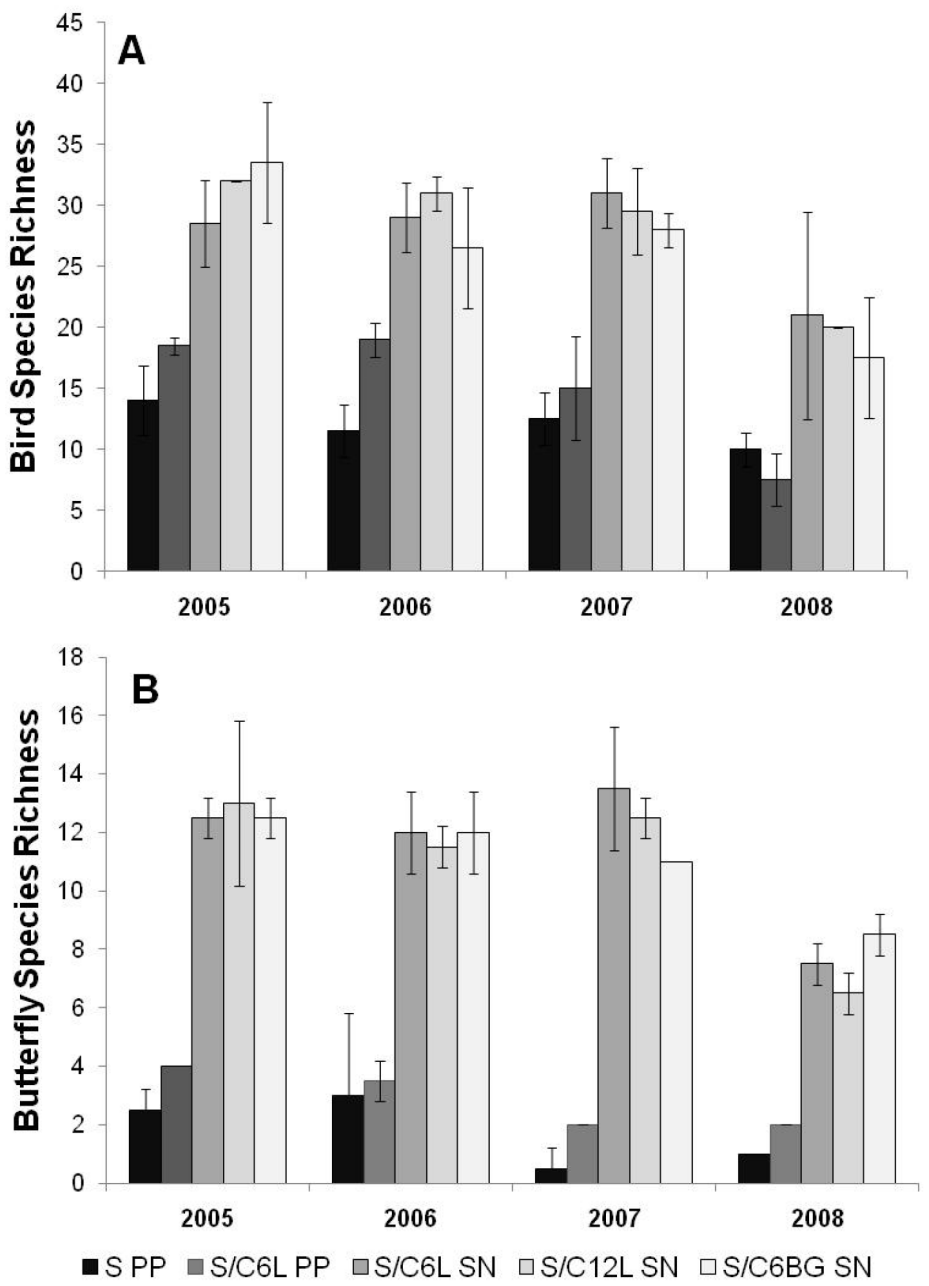
Effects of farmland management system on A) bird and B) butterfly species richness (±1 SD) 2005–08. See Table 1 for legend information. Butterfly surveys were conducted between May – September each year. Bird values are based on surveys between April – March each year (see text for details). Note: values for 2008 based on surveys between April – November 2008 only.

Over the 4-year experiment a total of 47184 birds (68 species) and 4896 butterflies (19 species) were recorded within the experimental systems. Full details of the species recorded on each treatment within each year are given in Tables S1 and S2 for birds and butterflies respectively. Both system and year were found to have a significant effect on bird (system *F*_4,35_ = 47.571, *P*<0.001; year *F*_3,32_ = 17.831, *P*<0.001) and butterfly (system *F*_4,35_ = 25.087, *P*<0.001; year *F*_3,32_ = 12.346, *P*<0.01) species richness, with systems that included SNRG having consistently higher species richness across years (Fig. 1). Approximately 25% of the bird (17/68) and butterfly (5/19) species recorded were observed only in SNRG, with 94% of all individual butterflies counted in this habitat. *Aphantopus hyperantus* (ringlet), *Pieris napi* (green-veined white) and *Maniola jurtina* (meadow brown) made up 66% of all butterfly individuals recorded. The flocking bird species *Corvus corone* (carrion crow), *Turdus pilaris* (fieldfare) and *Sturnus vulgaris* (starling) made up 71% of all individual birds recorded, with the latter species accounting for 56%.

An unavoidable limitation of our systems-based approach is that grazing area differed between treatments. We therefore calculated and compared species densities and found significant effects of system and year on bird and butterfly species density (all *P*<0.001), with bird species density consistently higher for S PP across years. To examine the effects on PP habitats only, we excluded species observed on SNRG from the analysis (i.e. 36% of total area) but still found a significant effect of system and year on bird (system *F*_4,35_ = 1.883, *P*<0.001; year *F*_3,32_ = 0.618, *P*<0.001) and butterfly (system *F*_4,35_ = 2.546, *P*<0.001; year *F*_3,32_ = 1.330, *P*<0.001) species density. Areas grazed solely by sheep had consistently lower species density than mixed sheep and cattle systems for butterflies, but higher species density for birds. We found no evidence that the system using BG cattle was any better for bird and butterfly species than those based on conventional cattle at the same stocking density.

## Discussion

Much of the previous management research with sheep and cattle in upland areas has concentrated on particular aspects of the production cycle. The comparatively few studies which have attempted to adopt a more systems-based approach have remained focussed on livestock performance. The current study simultaneously quantified productivity and environmental impact for a number of management options; testing assumptions made by both the farming industry and conservation groups regarding the value of different components within upland systems.

### Effect of Introducing Cattle Grazing

Herbage yields from improved upland swards can be over five times higher than from indigenous grasslands [29]. Consequently, maximising the efficiency of use of this component within upland systems is critical to increasing output of livestock while minimising the use of purchased inputs. Here we have shown that incorporating suckler cows and calves into a sheep-only system based on improved pastures leads to an increase in total output per unit area of PP. While this was brought about partly through the relatively greater weight gain of calves relative to sheep, it has also been influenced by the liveweight gain of the lambs being improved when mixed grazed with suckler cows and calves regardless of sheep:cattle ratio or duration of grazing [19]. While systems incorporating cattle were estimated to be associated with a higher total output of enteric methane, the methane emission intensities from some mixed systems, taking productivity into account, were lower. There was some suggestion that cattle performance declined over time, but this may be in part due to the cattle being more responsive to year-to-year variation in climatic conditions [19].

Although co-species grazing of cattle and sheep on improved upland pastures has the potential to improve livestock performance by increasing pasture use efficiency [30], for both species to benefit their grazing must remain complementary. It has been reported that improvements in lamb growth have been achieved at the expense of growing steer performance [30], suggesting that cattle are more sensitive than sheep to sward conditions and may be disadvantaged in situations where these two species become competitive. The current study with stock types more typical of those found in upland areas found no evidence that suckling calf performance was influenced by cattle:sheep ratio. However, it appears that increasing the contribution of the cattle to the overall stocking rate increases the overall output of animal product from the system without incurring higher methane emission intensities.

Additional advantages of mixed as opposed to single species grazing could include better matching of the animals’ seasonal energy requirements to herbage production and diversification of animal products. In addition, manipulation of the botanical composition of swards and a more balanced use of vegetation resources could in turn promote ecological stability and reduce the risk of landscape degradation [31].

### Effect of Incorporating Summer Grazing of SNRG by Cattle

For a variety of reasons semi-natural communities are protected from agricultural development. Typically, therefore, the only means of altering agricultural output from these swards is through manipulating the stocking rate, stock type or timing of stocking. On many upland farms grazing has been withdrawn from SNRG areas as declining stock numbers are focussed on better quality pasture. If left unmanaged, invasive grasses such as *M. caerulea* can become dominant over large areas to the exclusion of other plant species [32], and grazing can be crucial for maintaining both floristic and structural diversity within such swards [10]. Cattle are comparatively unselective grazers and are more willing to consume *M. caerulea* than sheep [33]. The associated habitat value is clearly demonstrated by the species richness of butterflies and birds supported by the SNRG areas of the current study. At the same time, however, the bird surveys revealed that the PP also supported large numbers of birds, particularly invertebrate feeders, at specific times of the year. Population studies are now required to predict long-term trends for these and other indicator species under different grazing systems.

Although calf performance was lower on the native pastures than the sown swards, the animals achieved commercially acceptable growth rates on vegetation which would commonly be considered unsuitable for productive stock [19]. The finding that such a level of performance can be achieved using commercial crossbred cattle conflicts with the perceptions of many upland farmers and their advisors. Within this study comparisons of output from the different systems have been carried out on a PP area basis, as SNRG components would not typically be considered a productive element within upland grazing systems. While their utilisation has generally become dependent upon agri-environment payments this study has demonstrated their potential value in terms of improving overall system productivity. Removal of the cattle from PP to graze the SNRG swards for around ten weeks reduced the overall requirement of the system for improved pastures, freeing these up for other activities such as the provision of home-grown forage for conservation as winter feed.

### Gaseous Pollutant Emissions

Crucially, the production benefits associated with incorporating summer grazing of SNRG were achieved without incurring a significant methane emissions penalty. The estimated enteric methane emissions of the LimX cattle were generally similar on the PP and SNRG, reflecting the timing of the grazing of the rough pasture coinciding with it being at its most nutritious. The lower daily rates of estimated methane emissions for the BG cattle were due to these animals having lower energy requirements, in keeping with the slow-growing nature of this breed [19]. However, the same animals had the highest methane emissions intensities (i.e. g methane per kg calf growth) for the same reason, because a greater proportion of energy intake was used for cow and calf maintenance requirements rather than growth.

While methane emission were broadly similar on the two pasture types, nitrogenous gaseous emissions are likely to have been lower on the PP. A significant source of the nitrogenous gaseous pollutants ammonia and nitrous oxide (another significant greenhouse gas) is urea-nitrogen deposited in the urine of grazing ruminants [34], [35]. The concentration and daily outputs of urine nitrogen by cattle and sheep depend on the diet composition, particularly on the concentration of protein [36] and its degradability in the rumen [37]. The release of nitrous oxide from urine patches depends largely on soil and climatic conditions [38], [39], but in the present study any effects due to these between the different systems types would have been minimal. Instead, any differences in ammonia and nitrous oxide emissions between systems would have been largely related to diet composition, and in particular the ratio of nitrogen to soluble carbohydrates in the diet. Increasing the ratio of water soluble carbohydrates to nitrogen in fresh grass has been shown to reduce the proportion of dietary nitrogen excreted in urine [40], [41]. The *Molinia* and PP swards tended to have similar crude protein concentrations, although the permanent pasture had higher concentrations of water soluble carbohydrates than the *Molinia* [19]. The proportion of dietary nitrogen consumed by animals grazing the permanent pasture and subsequently excreted in urine was therefore likely to be less than that excreted by the animals grazing the SNRG. Field research is now required to confirm these inferences and to quantify the extent to which GHG emissions from upland systems can be reduced by manipulating management guidelines.

### Effect of Incorporating a Traditional Breed of Cattle Rather than a Mainstream Breed

The BG calves were smaller than their LimX equivalents, in keeping with what would be expected of a native breed type. Although traditional cattle breeds such as the Belted Galloway are perceived as being particularly suited to conservation management there was little evidence from this study of them providing specific grazing benefits for grassland biodiversity. At the same time the production performance of these animals was substantially poorer than that achieved by the modern breed type, and the higher liveweight gains of the LimX cattle were estimated to be associated with lower methane emission intensities from the systems incorporating these animals. Thus, while traditional breeds may provide cultural and aesthetic value, choosing to graze these rather than modern breed types selectively bred for improved production performance could be associated with a net increased environmental burden.

## Conclusions

In this study we found that mixed upland grazing systems consisting of sheep and cattle grazing improved livestock productivity and reduced methane emissions relative to sheep only systems. Systems that also included SNRG consistently supported more species of birds and butterflies, and it was possible to incorporate bouts of summer grazing of these pastures by suckler cows to meet habitat management prescriptions without compromising cattle performance overall. We found no evidence that the system incorporating a cattle breed popular as a conservation grazer was any better for bird and butterfly species richness than those based on a mainstream breed, yet methane emissions from such a system were predicted to be higher. The results from this study have demonstrated that strategies which promote the inclusion of cattle plus the integration of rough grazing could improve both the overall economic and environmental sustainability of upland sheep farming systems. There is currently much debate concerning future land management in the hills and uplands across Northern Europe. The results of this study give a much needed evidence base for the development of policies relating to farming systems in these areas and related support payments.

## Supporting Information

Table S1 **Summary of bird species recorded on experimental plots within different upland grazing systems 2005–2008.**
(XLSX)

Table S2 **Summary of butterfly species recorded on experimental plots within different upland grazing systems 2005–2008.**
(XLSX)
